# Non-Invasive Transcutaneous Vagus Nerve Stimulation for the Treatment of Fibromyalgia Symptoms: A Study Protocol

**DOI:** 10.3390/brainsci12010095

**Published:** 2022-01-12

**Authors:** Andrés Molero-Chamizo, Michael A. Nitsche, Armin Bolz, Rafael Tomás Andújar Barroso, José R. Alameda Bailén, Jesús Carlos García Palomeque, Guadalupe Nathzidy Rivera-Urbina

**Affiliations:** 1 Department of Clinical and Experimental Psychology, University of Huelva, 21007 Huelva, Spain; rafan@dpsi.uhu.es (R.T.A.B.); alameda@uhu.es (J.R.A.B.); 2 Leibniz Research Centre for Working Environment and Human Factors, 44139 Dortmund, Germany; nitsche@ifado.de; 3 Department of Neurology, University Medical Hospital Bergmannsheil, 44789 Bochum, Germany; 4 tVNS Technologies GmbH, Ebrardstr. 31, 91052 Erlangen, Germany; armin@bolzandsons.com; 5 Department of the Histology, School of Medicine, Cadiz University and District Jerez Costa-N., Andalusian Health Service, 11003 Cádiz, Spain; jesusc.garcia.sspa@juntadeandalucia.es; 6 Psychology Center, Faculty of Administrative Sciences, Autonomous University of Baja California, Ensenada 22890, Mexico; nathzidy.rivera@uabc.edu.mx

**Keywords:** chronic pain, fibromyalgia, transcutaneous, vagus nerve stimulation

## Abstract

Stimulation of the vagus nerve, a parasympathetic nerve that controls the neuro-digestive, vascular, and immune systems, induces pain relief, particularly in clinical conditions such as headache and rheumatoid arthritis. Transmission through vagal afferents towards the nucleus of the solitary tract (NST), the central relay nucleus of the vagus nerve, has been proposed as the main physiological mechanism that reduces pain intensity after vagal stimulation. Chronic pain symptoms of fibromyalgia patients might benefit from stimulation of the vagus nerve via normalization of altered autonomic and immune systems causing their respective symptoms. However, multi-session non-invasive vagal stimulation effects on fibromyalgia have not been evaluated in randomized clinical trials. We propose a parallel group, sham-controlled, randomized study to modulate the sympathetic–vagal balance and pain intensity in fibromyalgia patients by application of non-invasive transcutaneous vagus nerve stimulation (tVNS) over the vagal auricular and cervical branches. We will recruit 136 fibromyalgia patients with chronic moderate to high pain intensity. The primary outcome measure will be pain intensity, and secondary measures will be fatigue, health-related quality of life, sleep disorders, and depression. Heart rate variability and pro-inflammatory cytokine levels will be obtained as secondary physiological measures. We hypothesize that multiple tVNS sessions (five per week, for 4 weeks) will reduce pain intensity and improve quality of life as a result of normalization of the vagal control of nociception and immune–autonomic functions. Since both vagal branches project to the NST, we do not predict significantly different results between the two stimulation protocols.

## 1. Introduction

Fibromyalgia is a disabling chronic disease. In addition to chronic and generalized pain, the syndrome includes extreme fatigue, insomnia and other sleep disorders, affective and emotional disorders, such as depression and anxiety, cognitive impairment, paresthesia, allergy symptoms, joint stiffness, tendinopathy, neuropathic and rheumatologic symptoms, chemical and skin hypersensitivity, irritable bowel syndrome, and other symptoms whose common etiopathogenesis is unknown [1,2,3]. Because of the unknown biological mechanisms of fibromyalgia, current pharmacological therapies are aimed at alleviating its symptoms, and the efficacy of available treatments is limited [4]. Moreover, generalized pain and extreme fatigue are usually refractory to any therapeutic attempt [4,5].

The parasympathetic vagus nerve (the 10th cranial nerve) innervates multiple internal organs and integrates sensory, motor, and autonomic information by four vagal nuclei. The primary central relay of vagal afferents is the nucleus of the solitary tract (NST) in the brainstem, and several branches of this nucleus project to different brain areas [6]. The myelinated A- and B-fibers of the vagal nerve send somatosensory, motor, and autonomic signals [7], and the complex system of vagal projections is involved in inflammatory, immune, nociceptive, and emotional processes [8,9]. A relevant role of the vagal nerve in pain processing is the transmission of peripheral inflammation signals to the central nervous system [10]. Additionally, neuroimaging studies have identified neuromodulatory activity in areas related to the sensory and emotional processing of pain, such as the insular cortex and anterior cingulate cortex, after vagal stimulation [11], which reveals that the central system of pain processing receives vagal afferences. Due to the multi-system control of the vagal nerve [12], some clinical conditions refractory to conventional treatment, such as epilepsy, depression, and inflammatory diseases, have been shown to benefit from invasive stimulation of this nerve [13]. Recent clinical and experimental studies have shown that stimulation of the vagus nerve also has therapeutic potential in other diseases, including pain syndromes [14,15]. Vagus nerve stimulation is a Food and Drug Administration (FDA)-approved somatic treatment for different pathologies. The precise mechanisms through which vagal stimulation induces clinical improvements are not well understood in detail [16,17], but regulation of the autonomic and immune systems and a specific effect on chemical mediators of inflammation are relevant physiological mechanisms of this intervention [18].

Considering that the vagus nerve controls pain signals towards the central nervous system [14,19,20], and growing evidence of autonomic dysfunction in fibromyalgia in terms of sympathetic hyperactivity [21,22,23,24,25,26,27], it can be argued that rebalancing the sympathetic–parasympathetic activity via vagal stimulation might reduce the intensity of pain and stabilize the autonomic symptoms of this disease. In addition, an immune system alteration [28] seems to mediate the interaction between the autonomic nervous system and the chronic inflammatory state in fibromyalgia, apparently by the influence of inflammatory cytokines and chemokines [28,29,30]. This well-described inflammatory state in fibromyalgia patients is critically important for treatment approaches. Auricular and cervical vagus nerve stimulation might reduce such inflammatory overactivity as well via efferent modulation of the activity of the spleen and inflammatory processes [18] and hereby reduce pain symptoms [19]. In accordance, invasive vagal nerve stimulation has been shown to be effective in treatment-resistant fibromyalgia patients, with a tolerability profile similar to that described for the treatment of epilepsy and depression [31]. However, to the best of our knowledge, the potential of non-invasive auricular and cervical vagus nerve stimulation under repeated sessions to improve fibromyalgia symptoms has not been evaluated in randomized, sham-controlled clinical studies. Auricular and cervical transcutaneous vagus nerve stimulation (tVNS), two non-invasive low-intensity electric stimulation procedures [32,33], have however been explored with respect to their potential to modulate the activity of this nerve in other clinical settings [20,34,35,36,37], and the effectiveness of these non-invasive methods might not differ significantly from those of invasive stimulation, while non-invasiveness provides an advantageous safety profile [14,19,38,39,40]. 

Since fibromyalgia involves a dysregulation of the autonomic (high sympathetic tone) and immune (enhanced pro-inflammatory activity) systems [25,41], non-invasive tVNS is expected to improve the symptoms of fibromyalgia, including chronic, musculoskeletal, and generalized pain [42,43], through modulation of the vegetative and immune systems [44]. The main objective of this intervention study is to evaluate the effects of repeated sessions of tVNS on pain and other symptoms in fibromyalgia patients, comparing auricular and cervical tVNS protocols which have been probed in other pathologies [45,46,47,48,49]. According to the effectivity reported in these studies, we hypothesize that a multi-session auricular and cervical tVNS protocol carried out under safety standards will provide analgesic and therapeutic effects in fibromyalgia patients, in comparison with sham and axillary nerve stimulation controls, and secondarily will also normalize the autonomic (via heart rate variability) and immune (via pro-inflammatory cytokine levels) functions supposedly altered in this disease.

## 2. Materials and Methods

### 2.1. Participants

Adult fibromyalgia patients, in an age range between 18 and 69 years, recruited from the Hospital Juan Ramón Jiménez of Huelva, Spain, will be interviewed to voluntarily participate in this monocentric study. Inclusion criteria will be the diagnosis of fibromyalgia by a physician, based on the 2010 American College Rheumatology (ACR) fibromyalgia diagnostic criteria, and the maintenance of characteristic symptoms for more than a year. A moderate to high pain intensity according to analog pain scales (above 4 points over 10), maintained for more than 6 months, will also serve as an inclusion criterion. The usual individual medical treatment for fibromyalgia will be maintained throughout the study, including psychopharmacological treatments. All genders will be included. Exclusion criteria will include standard conditions to be considered for VNS (cardiac arrhythmia, sleep apnea, pregnancy, etc.) [49], other chronic diseases producing widespread pain not related to known symptoms of fibromyalgia, multiple pharmacological treatments for symptoms different from those of fibromyalgia, primary psychiatric syndromes (psychosis, bipolar disorder, etc.) with or without treatment via central nervous system-acting medication, drug dependence (including smoking), vagal syndrome or history of vagal symptoms, and chronic pain and fibromyalgia secondary to inflammatory rheumatic diseases. Previous interventions with non-invasive brain stimulation methods or invasive VNS will also constitute exclusion criteria. An electrocardiogram will be performed in all participants before the study. Participants with arrhythmia and other cardiac alterations will be excluded from the study.

Sample size calculation will be based on the hypothesized significant differences between (auricular vs. cervical vs. sham vs. axillary) and within (baseline vs. post-intervention) groups. We conducted an a priori calculation of the sample size by the GPower statistical power analysis tool (3.1.9.2) for a mixed-model repeated-measures ANOVA (with groups and pre/post-intervention measures as levels of the between-group and within-group factors) [50], as a primary outcome parameter conducted for the pain questionnaire (BPI). Because the main parameter of interest for this calculation is the interaction between the factors group and measure, we conducted the analysis for the within–between interaction, setting the following input parameters: effect size (f) = 0.2 (medium size), α value = 0.05, Power (1-ß) = 0.95, number of groups = 4 (pooled sham, axillary stimulation, cervical tVNS, and auricular tVNS), number of measurements = 2 (baseline as pre-intervention, and at the end of the intervention), correlation for repeated measures = 0.5, and with nonsphericity correction (1). The output parameters were: non-centrality parameter = 17.92, critical F = 2.68, actual power 0.951, and total sample size = 112. Taking into account these estimates and adding 20% to the calculated sample to compensate for possible dropouts, we will include 34 patients per group (total sample size of 136).

Written informed consent (available in Supplemental material) will be provided by all patients prior to their participation in the study. The clinical trial will be submitted for consideration and approval by the Regional Ethics Committee for Clinical Studies. In accordance with national legislation (93/42/EEC and RD 1090/2015), the study will be pre-registered in the Spanish registry of clinical studies (https://reec.aemps.es/reec/public/web.html#, accessed on 28 December 2021). The study complies with the code of Ethics of the World Medical Association Declaration of Helsinki (amend 64th, 2013).

### 2.2. tVNS

**Figure 1:**
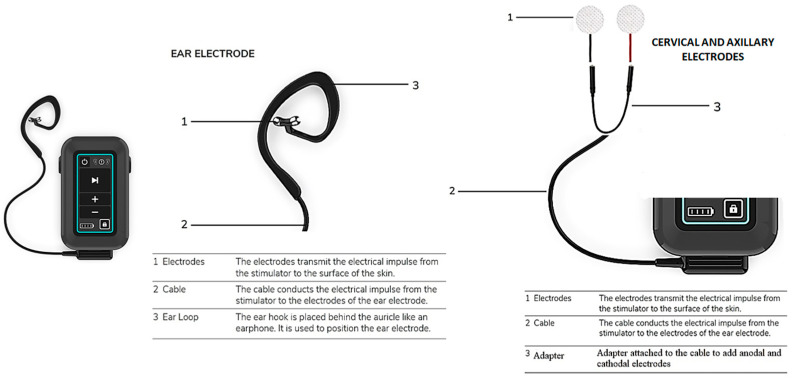
tVNS^®^ *L* device for transcutaneous stimulation of the vagus nerve.

**Figure 2:**
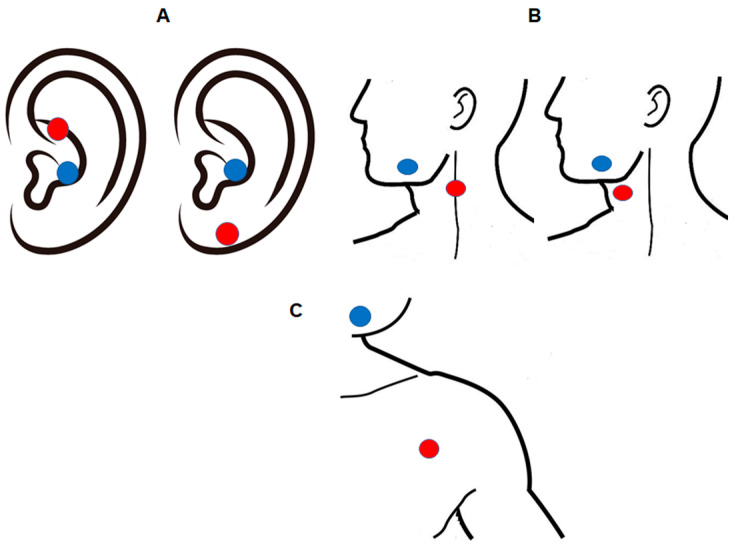
Non-invasive transcutaneous auricular (**A**) and cervical (**B**) vagus nerve stimulation will be applied to evaluate the effect of this intervention on fibromyalgia symptoms. Three different electrode montages will be tested: (**A**) anode positioned over the cymba conchae of the left auricle (verum stimulation) or the left ear lobe (sham) and cathode (return electrode) over the antitragus, (**B**) anode positioned over the left cervical branch of the vagus nerve in the neck (adjacent to the carotid) (verum stimulation) or two centimeters anterior to this branch (sham) and cathode below the cheek, (**C**) anode over the left axillary nerve and cathode below the cheek (only verum stimulation, as an added control group). The red circles represent the anode electrode, and the blue circles represent the cathode electrode.

Auricular vagal stimulation will be performed by the tVNS^®^ *L* device (tVNS Technologies GmbH, Erlangen, Germany, https://u.pcloud.link/publink/show?code=kZ0mYfXZRXxQqurVY9SB8okpGtAc55aX7nrX, accessed on 28 December 2021). tVNS^®^ *L* is a battery-driven electrical stimulator connected to an ear electrode which is positioned over the skin of the cymba conchae (Figure 1). Thus, auricular vagal stimulation will be applied using the typical ear electrodes of the tVNS^®^ *L* device. A technical variant of this device, in which the original connector is modified to incorporate self-adhesive electrodes, will be used for cervical vagal stimulation and axillary nerve stimulation (the latter as an added control group). Thus, stimulation over the cervical branch of the vagus nerve and axillary nerve stimulation will be applied by adapted electrodes for transcutaneous stimulation outside the ear (Figure 1). In order to effectively access cervical vagal projections, as reported in previous studies [36], 2 cm^2^ self-adhesive anodal and cathodal electrodes will be used for cervical vagal stimulation. These adapted electrodes will also be used in the active control group with axillary nerve stimulation (Figure 2). Since efferent fibers of the vagus nerve controlling cardiac functions are mainly located in the right branch [51], but no arrhythmic effects have been reported after left transcutaneous vagus nerve stimulation [52], tVNS will be applied over the left branch for safety reasons. For sham auricular tVNS, the anodal electrode will be placed over the center of the left ear lobe as previously reported [53], because there is no vagal innervation around the ear lobe [54] and stimulation of this area does not alter brain activity, including the brain stem [55]. The cathode will be placed over the antitragus. (Figure 2A). Accordingly, in the sham cervical tVSN protocol, the anodal electrode will be placed 2 cm anterior to the cervical branch of the vagus nerve, and the cathode below the cheek (Figure 2B). This method and these stimulation parameters have been previously used for reliable blinding in other clinical studies [56]. Series of electrical pulses with 250 μs pulse width, 25 Hz frequency, and 28 s inter-burst interval (32 s on/28 s off duty cycle) will be applied in each intervention session to induce effective vagal stimulation and avoid stimulation habituation. Auricular tVNS will be applied at 1mA intensity for 30 min per session (1 session per day, 5 consecutive days a week, 20 sessions in total). In order to reach equivalent current density as for the auricular electrodes, which include a maximum contact area of 1 cm^2^, the stimulation intensity for non-auricular electrodes (2 cm^2^) will be adjusted to 2 mA, which will allow achieving comparable current densities at the electrode–skin interface for the respective electrode sizes. The other non-auricular stimulation parameters will remain the same as for the auricular stimulation protocol.

### 2.3. Outcome Measures

The intensity and magnitude of the baseline symptoms and primary (pain) and secondary (fatigue and health-related quality of life, sleep disorders, depression, physiological measures) outcome measures will be recorded by a validated Spanish version of the Brief Pain Inventory (BPI) [57] (pain intensity measure), the Patients’ Global Impression of Change (PGIC) [58] (restricted to pain intensity changes), a validated Spanish version of the revised Fibromyalgia Impact Questionnaire (FIQR) [59] (fatigue and health-related quality of life, as a secondary measure of general symptom improvement), the Pittsburgh Sleep Quality Index (PSQI) [60,61] (sleep measure), and the Beck Depression Inventory (BDI) [62,63,64,65] (depression measure). Additionally, heart rate variability (the high/low heartbeat ratio as an index of cardiac vagal regulation) and pro-inflammatory cytokine levels (TNF α, IL-1β, IL-6) will be recorded as secondary physiological outcome measures. Heart rate variability will serve as an indirect marker of the efficacy of vagal neuromodulation, at the level of its autonomic effects.

### 2.4. Randomization Method

The tVNS effects on fibromyalgia symptoms will be evaluated in a randomized, prospective, double-blind, sham-controlled, and parallel group clinical trial, with a mixed between-group and within-group design. The data of the recruited participants will be included in a secure database, and a code per group for randomization will be generated. To allocate subjects to each group with complete randomness and independence regarding the intervention, simple randomization will be applied [66], by which the participant codes will be assigned to the respective groups via a 1:1 randomization procedure (equal allocation) [67,68] using the computer spreadsheet Microsoft Excel.

### 2.5. Procedure

The patients will be recruited according to the inclusion and exclusion criteria. Then, only patients without alterations in the electrocardiogram will be randomly assigned to one of five groups: auricular vagus nerve stimulation at 25 Hz frequency (atVNS) (*n* = 34); sham auricular vagus nerve stimulation (Sham atVNS) (*n* = 17); cervical vagus nerve stimulation at 25 Hz frequency (ctVNS) (*n* = 34); sham cervical vagus nerve stimulation (Sham ctVNS) (*n* = 17); axillary nerve stimulation at 25 Hz frequency (ANS) (*n* = 34). Pre-intervention baseline measures (FIQR, BPI, PSQI, and BDI) will be performed once a week for 3 weeks and before the first intervention day, resulting in four baseline measures (one per week) to evaluate the stability of the symptoms. Then, each participant will receive five 30 min consecutive sessions of stimulation (verum or sham) per week (one per day), for four weeks (in total, 20 sessions) over the left branch of the auricular or cervical vagal nerve or over the left axillary nerve, depending on the group. The patients will not be aware of the stimulation condition (verum vs. sham) within each group, and data analyses will be conducted by researchers not involved in the respective interventions, who will use numerical codes for each experimental condition in order to assure double blinding. Once the analyses have been conducted, the codes will be revealed. In the atVNS verum and sham groups, the anodal electrode will be positioned over the cymba conchae of the left auricle and the center of the left ear lobe, respectively, and the cathode (return electrode) over the antitragus. In the ctVNS verum group, the anodal electrode will be positioned on the surface of the throat that corresponds to the position of the left cervical branch of the vagus nerve, adjacent to the position of the cervical carotid artery (2 cm anterior to this branch for the sham ctVNS condition), and the cathode over the lower cheek. In the ANS verum group, the anode will be positioned over the left axillary nerve under the shoulder, and the cathode over the lower cheek. The positions of the electrodes for the respective procedures are shown in Figure 2.

**Figure 3:**
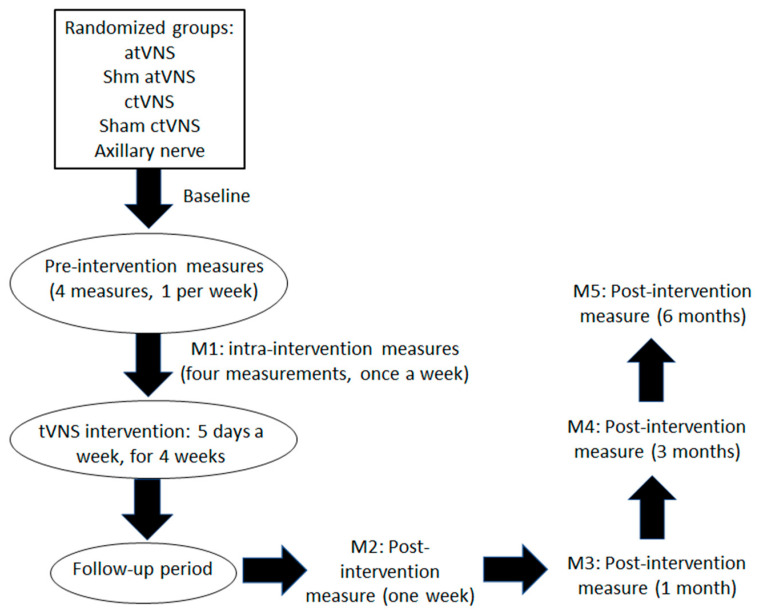
Study design. Measurements refer to primary and secondary outcome measures (FIQR, BPI, PGIC, PSQI, and BDI scores). Heart rate variability will be recorded before and after each tVNS session, and pro-inflammatory cytokine levels (TNF α, IL-1 β, IL-6) will be analyzed before the start of the intervention and after 10 and 20 intervention sessions, as well as 1 week, and 1 month after the end of the intervention. ANS, axillary nerve stimulation at 25 Hz frequency; atVNS, auricular vagus nerve stimulation at 25 Hz frequency; ctVNS, cervical vagus nerve stimulation at 25 Hz frequency; Sham atVNS, auricular vagus nerve stimulation at 1 Hz frequency; Sham ctVNS, cervical vagus nerve stimulation at 1 Hz frequency.

The post-baseline outcome measures (FIQR, BPI, PGIC, PSQI, and BDI) will be recorded immediately after the fifth session of each intervention week, resulting in four intra-intervention measurements. Four additional post-intervention follow-up measures will be recorded to evaluate potential short- and long-lasting treatment effects, a week, a month, 3, and 6 months after the last intervention session, resulting in four intra-intervention and four post-intervention measurements. Although no conclusive evidence about tVNS effects on heart rate variability is available [69], heart frequency will be recorded before and after each tVNS session, the high-frequency/low-frequency heartbeat ratio will be calculated, and data will be correlated with the outcome measures. Pro-inflammatory cytokine levels taken from blood samples before the start of the intervention and immediately after 10 and 20 intervention sessions, as well as 1 week, and 1 month after the end of the intervention, will be analyzed as proposed biomarkers of the anti-inflammatory effects of the intervention [70]. To measure plasma cytokine levels, the enzyme-linked immunosorbent assay (ELISA) protocol will be used [71]. Plasma collection will be selected to optimize the yield of cytokines of interest (TNF α, IL-1β, IL-6) [72]. Within 1 h after collection, blood will be centrifuged for 10 min at 1300 g and 4 °C, and the supernatant will be transferred to a separate tube and then centrifuged for 10 min at 16,000 g and 4 °C. The resulting platelet-poor plasma will be stored in 0.5 mL aliquots at low temperatures (−80 °C) in order to maintain cytokine stability until the analysis. After each intervention, participants will be asked to guess the stimulation condition (real, sham, or unable to guess) for evaluation of successful blinding as previously reported [73], and any adverse effect of the stimulation or any aspect related to tVNS will be evaluated via an adapted standard questionnaire for assessment of adverse effects induced by a related non-invasive brain stimulation tool, namely, transcranial direct current stimulation (tDCS) [74], as a measure of the safety of the intervention. All sessions will take place at the aforementioned hospital. Figure 3 depicts the design of the study.

### 2.6. Statistical Analysis

To exclude differences of baseline measurements (BPI scores as primary outcome measure, and FIQR, PGIC, PSQI, and BDI scores as secondary outcome measures) between groups, a mixed model 4 × 4 ANOVA will be used, with one between-group factor with four levels (4 groups), and one within-subject factor (four baseline measurements). A mixed model 4 × 8 ANOVA, with one between-group factor with four levels (four groups) and one within-subject factor (8 measurement days), will be used to analyze the scores of the outcome measures obtained in each of the questionnaires throughout the study (intra-intervention and follow-up period). In case of significant results of the ANOVAs, exploratory Bonferroni-corrected post-hoc *t*-tests will be conducted. A univariate analysis by a Chi-square test will be performed to explore the success of blinding with regard to the intervention, and *t*-tests as well as Chi-square tests will be conducted to exclude demographic differences (gender, age, and years of education) between groups. Pearson’s correlation coefficient (Pearson’s *r*) will be calculated for each tVNS group to analyze possible correlations between vagal activity measures (heart rate variability) during the intervention and questionnaire scores (FIQR, BPI, PGIC, PSQI, and BDI), as well as between pre- and post-intervention pro-inflammatory cytokines levels and outcome measures. The critical level of significance for all statistical analyses will be set to *p* < 0.05. The analyses will be carried out using the SPSS statistical software package.

## 3. Discussion

This study aims to explore the effects of multiple tVNS sessions on fibromyalgia symptoms. Because the vagus nerve modulates afferent pain signals to the central nervous system and the tone of the autonomic, immune, and emotional systems [19], the application of tVNS, a non-invasive low-intensity electric stimulation technique with potential to activate A- and B-fibers [75], might normalize the functions of vagal afferences, which seem to be pathologically altered in fibromyalgia [25], and thus reduce the characteristic symptoms of this disease, particularly muscular and generalized pain. Encouraging results have been reported for invasive vagal stimulation procedures in fibromyalgia treatment [31], but controlled clinical trials using non-invasive vagal stimulation are missing with respect to the effectiveness and safety of this neuromodulation option. The proposed study might shed light on the involvement of vagal activity and sympathetic–vagal balance in chronic pain and other symptoms of patients with fibromyalgia and also provide direct comparisons between two tVNS protocols in terms of effectiveness and safety. In particular, we hypothesize that this protocol of vagal neuromodulation is effective and safe for improving pain and other symptoms in these patients. The inclusion of a sham intervention condition is important to demonstrate the specificity of the therapeutic effectivity of this intervention. The inclusion of an active control condition, in which a nerve not related to fibromyalgia symptoms (the axillary nerve) is stimulated, will deliver further evidence about the specificity of the effects. The high number of intervention sessions (20 sessions, over 4 weeks) included in this stimulation protocol might serve to control for null effects of tVNS due to under-dosing.

The results of this intervention will help to elucidate if tVNS is a feasible method to induce clinically relevant analgesic effects in fibromyalgia patients (primary outcome measure). In addition, considering that the vagus nerve controls the autonomic nervous system and inflammatory signals [18,19,20], whose activity is apparently altered in fibromyalgia patients [25,27], other symptoms, including sleep and mood disorders, and quality of life (secondary outcome measures) could also benefit from this intervention. However, this study might face some limitations. First, high inter-individual variability in the fibromyalgia symptoms [76] might make it difficult to evaluate the efficacy of the treatment, despite the control of the baseline symptom state. Future multi-center studies with highly representative samples might be required to support the validity of the results of the envisaged pilot study. Preliminary results accomplished by this study may also be extended in future studies by the inclusion of other groups with chronic pain not primarily related to disturbances of the autonomic or immune systems, such as, for example, post-stroke pain, in which muscle pain symptoms are due to central alterations.

## 4. Conclusions

We have described a non-invasive vagal nerve stimulation protocol aimed to ameliorate chronic pain and improve quality of life in fibromyalgia patients. The results of this study will also provide information about the effectiveness and safety of two different vagal nerve stimulation protocols. In addition, this study protocol will allow to attribute a therapeutic effect of tVNS to the specific neuromodulation of the vagus nerve because of the inclusion of a control group with non-vagal stimulation. Preliminary findings from this pilot study will provide valuable information to design future clinical trials and systematic studies to optimize the therapeutic outcomes of tVNS.

## Data Availability

Not applicable.

## Supplementary Materials

The following supporting information can be downloaded at: https://www.mdpi.com/article/10.3390/brainsci12010095/s1, Supplementary 1 (S1)—Participants: Informed consent; Supplementary 2 (S2)—Participants: Procedure and objectives of the study; Supplementary 3 (S3)—Participants: Exclusion criteria.
