# Supplementary material for: Non-Invasive Transcutaneous Vagus Nerve Stimulation for the Treatment of Fibromyalgia Symptoms: A Study Protocol
Source: Brain Sci. 2022 Jan 12;12(1):95. doi: 10.3390/brainsci12010095 (PMC8774206; doi:10.3390/brainsci12010095)
Supplement: Supplementary file 1 [file brainsci-12-00095-s001.zip › brainsci-1555557-supplementary.pdf]

Non-invasive transcutaneous vagus nerve stimulation for treatment of fibromyalgia symptoms: A study protocol

Andrés Molero-Chamizo[1*], Michael A. Nitsche[2,3], Armin Bolz[4], Rafael Tomás Andújar Barroso[1], José R. Alameda Bailén[1], Jesús Carlos García Palomeque[5], Guadalupe Nathzidy Rivera-Urbina[6]

[1]University of Huelva. Department of Clinical and Experimental Psychology. Huelva, Spain

[2]Leibniz Research Centre for Working Environment and Human Factors, Dortmund, Germany (https://orcid.org/0000-0002-2207-5965)

[3]Department of Neurology, University Medical Hospital Bergmannsheil, Bochum, Germany

[4]tVNS Technologies GmbH, Ebrardstr. 31, Erlangen, Germany (www.t-vns.com)

[5]Histology Department. School of Medicine, Cadiz University and District Jerez Costa-N., Andalusian Health Service, Spain

[6]Autonomous University of Baja California, México (http://orcid.org/0000-0001-8102-4992)

[*]**Corresponding author**:

Andrés Molero-Chamizo (https://orcid.org/0000-0003-0381-8927)

University of Huelva. Department of Clinical and Experimental Psychology, Psychobiology Area.

Campus El Carmen. 21071 Huelva, Spain

Telephone number (34)959218478

E-mail: andres.molero@dpsi.uhu.es

Supplemental Material

Table of Contents

Supplementary 1 (S1) – Participants: Informed consent

Supplementary 2 (S2) – Participants: Procedure and objectives of the study

Supplementary 3 (S3)– Participants: Exclusion criteria

S1

INFORMED CONSENT

The principal researcher of the project **Non-invasive transcutaneous vagus nerve stimulation for treatment of fibromyalgia symptoms: A study protocol**

has informed to Mr/Mrs………………. ………………… with Official Identification …….. …………., on the general procedure of this study and its purpose,

and Mr /Mrs, ………………………………… in knowledge of his/her rights as volunteer participant about the possibility and the moment in which he/she can revoke his/her participation, and the measures taken for the protection of personal data according to current regulations (Organic Law 15/1999, December 13, Protection of Personal Data: Consolidated text, last modified March 5, 2011),

PROVIDES his/her consent to participate in the current research, which follows the 2013 Declaration of Helsinki principles and is designed in accordance with current regulations (National Law 14/2007 on Biomedical Research and Law 14/2011 on Science, Technology and Innovation, with regard to research procedures with human beings).

Signed: Andrés Molero Chamizo

Signature as Principal Researcher

Participant signature

POSSIBILITY OF LEAVING THE STUDY VOLUNTARILLY

I revoke the consent given on the date …………………… .., and I do not wish to continue my participation in the study

Participant signature

Huelva . . . ., 2022

S2

PROCEDURE AND OBJECTIVES OF THE STUDY

Non-invasive transcutaneous vagus nerve stimulation for treatment of fibromyalgia symptoms: A study protocol

Objectives:

1. To evaluate the effects of repeated sessions of transcutaneous vagus nerve stimulation (tVNS) on pain, quality of life and other symptoms in fibromyalgia patients, including autonomic and immune system related-symptoms, comparing auricular and cervical tVNS protocols which are effective in the treatment of other pathologies.

2. Evaluate the maintenance of the possible effects in the medium and long term.

Expected benefits:

Possible improvement of the autonomic and pain symptoms characteristic of this disease by a non-pharmacological treatment, which is safe, non-invasive and painless.

Potential undesirable effects:

The stimulation procedure of this study respects human dignity and is designed in accordance with current regulations. There is scientific evidence of therapeutic effects of tVNS in clinical practice and its use can provide significant information in clinical and basic research (Farmer et al., 2021). In order to fully preserve the integrity of the participants, the stimulation parameters that we will use will conform to the published safety recommendations regarding the application of tVNS (Komisaruk and Frangos, 2022). Under these recommendations, no serios undesirable effects are expected.

Being informed of the experimental procedure that will be carried out in this study, and the possibility to revoke consent to participate at any time as volunteer participant, Mr / Mrs.
…………………………………………………………………………………………
……., with Official identification ……. …………………………………,

PROVIDES consent to participate in this research.

Signature of the participant

Signature of the principal researcher: Andrés Molero Chamizo

Huelva, …………………… ..2022

S3

EXCLUSION CRITERIA

Non-invasive transcutaneous vagus nerve stimulation for treatment of fibromyalgia symptoms: A study protocol

As volunteer participant in this study, I declare that:

1. I do not have the diagnosis of cardiac arrhythmia or other cardiac alterations, epilepsy, stroke, sleep apnea, or neurological diseases.

2. I do not have the diagnosis of any disease producing widespread pain not related with fibromyalgia, nor chronic pain and fibromyalgia secondary to inflammatory rheumatic diseases.

3. At present, I do not have pharmacological treatments for symptoms different from those of fibromyalgia.

4. I do not have the diagnosis of primary psychiatric diseases (psychosis, bipolar disorder, depression, etc.) and I am not taking central nervous system-acting medication for these diseases.

5. I have no drug dependence (including tobacco or alcohol dependence).

6. I do not have vagal syndrome or history of vagal symptoms.

7. I had no previous interventions with non-invasive brain stimulation methods or invasive VNS.

8. I do not carry any metallic implant in my head, nor any internal pacemaker or metallic medical device.

9. For women: I am not pregnant.

Researcher signature

Participant signature

Huelva, ……………………………………, 2022
